# Supplementary material for: Synthetic virology approaches to improve the safety and efficacy of oncolytic virus therapies
Source: Nat Commun. 2023 May 26;14:3035. doi: 10.1038/s41467-023-38651-x (PMC10213590; doi:10.1038/s41467-023-38651-x)

Synthetic virology approaches to improve the safety and efficacy of oncolytic virus therapies

Azad et al.

Supplementary Information

Supplementary Figure 1

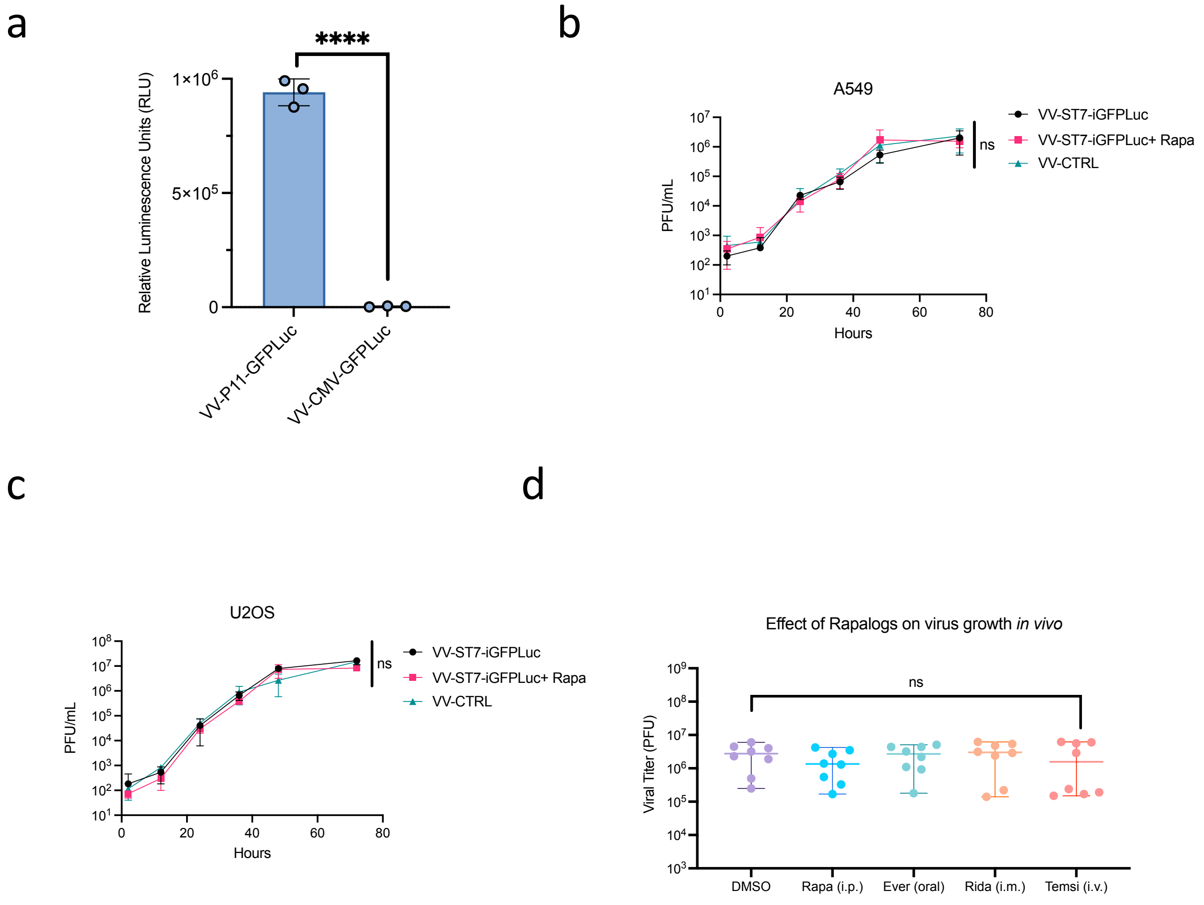

Supplementary Figure 1. (a) Compared to the P11 vaccinia virus promoter, the CMV promoter in vaccinia virus exhibited significantly lower activity. **(b-c) The ST7 switch, and rapamycin have no significant effects on virus growth kinetics** Multistep growth curves of VV-ST7-iGFPLuc compared to control vaccinia virus at different time points from (**b)** A549 or **(c)** U2OS cells infected at MOI 0.01 in the presence or absence of 10 nM rapamycin. (**d**) Quantitation of viral titers within HT-29 tumors from CD-1 nude mice 10 days after Rapalogs were introduced into the mice. Tumors were infected with VV-ST7-iGFPLuc at 1E7 PFU/tumor prior to the introduction of Rapalogs. Data indicate means ± SD of three biological replicates. ns $P > 0.05$, *$P < 0.05$ **$P < 0.003841$, ***$P < 0.000125$, ****$P < 0.001$ in unpaired two-samples t-test.

Supplementary Figure 2

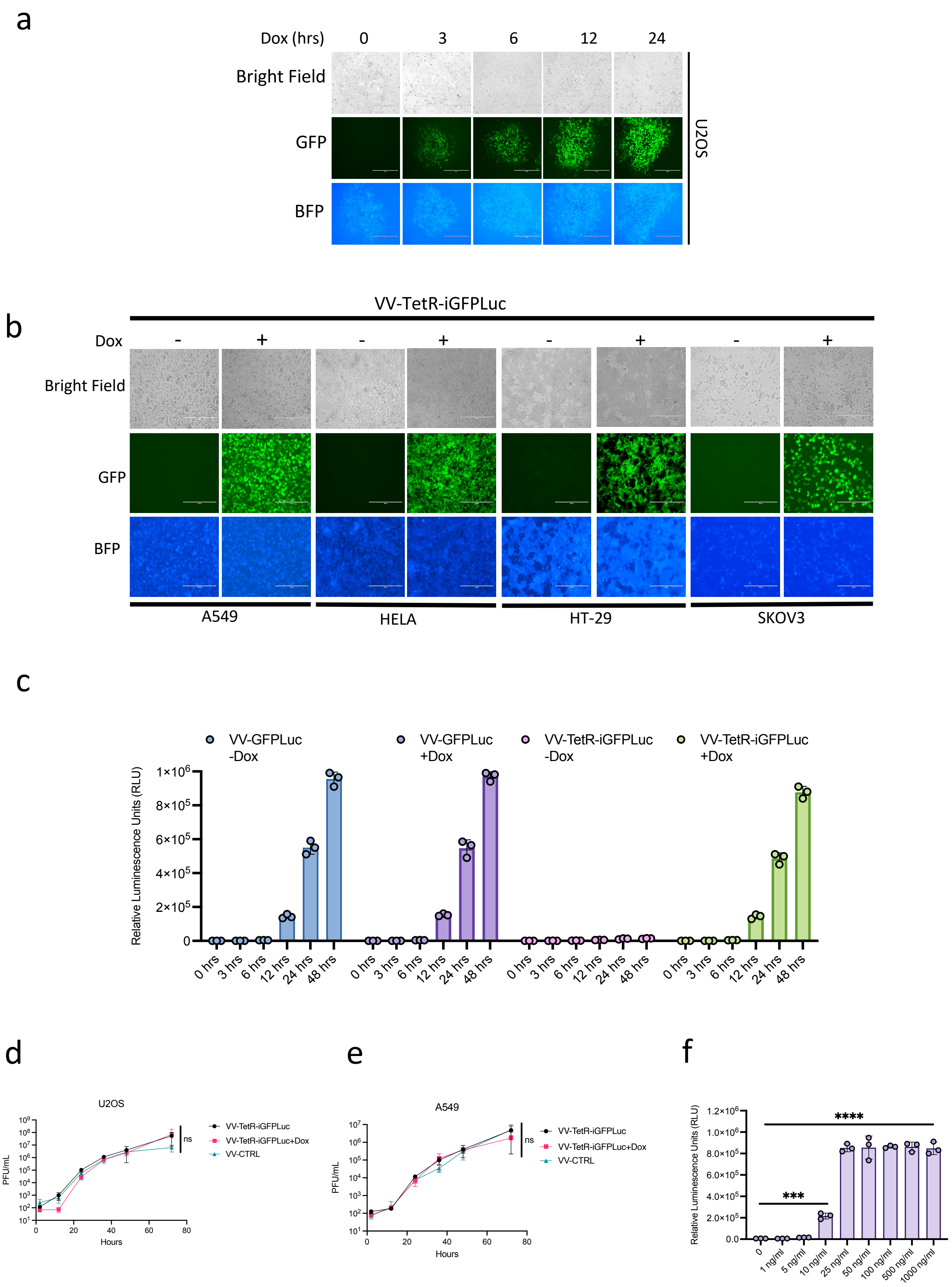

Continuation of Supplementary Figure 2

g

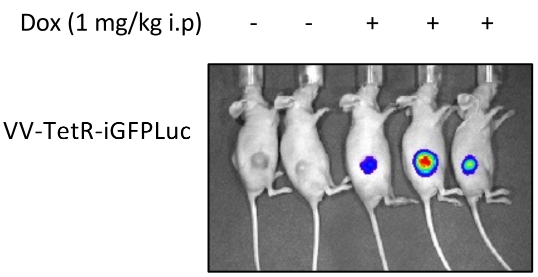

h

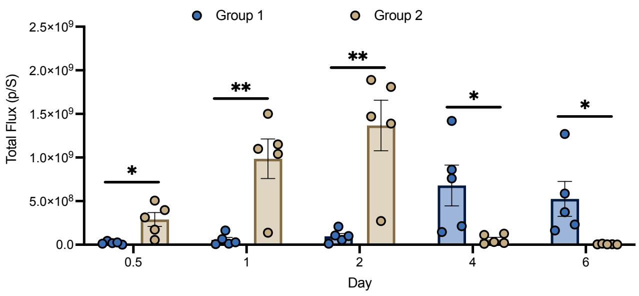

i

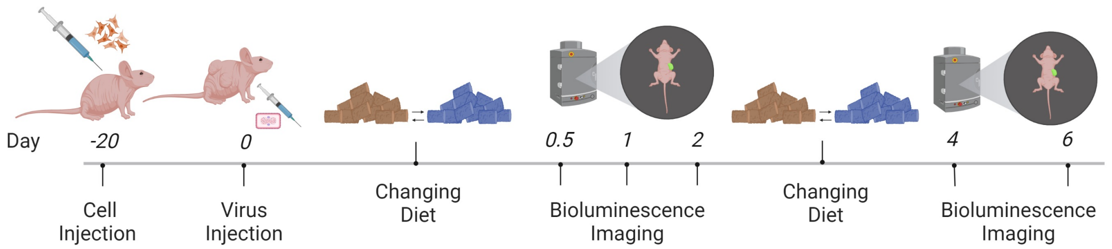

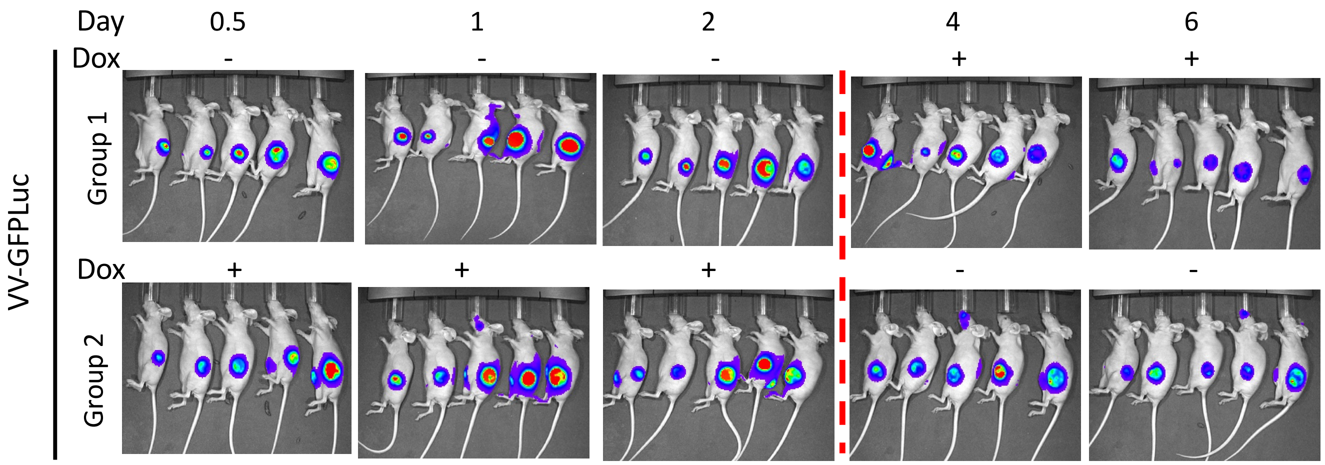

j

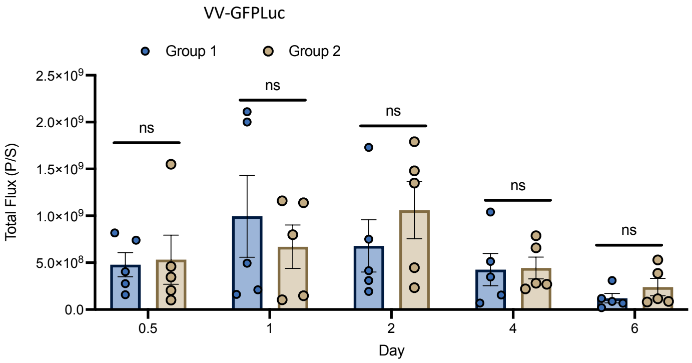

Supplementary Fig 2. *In vitro* and *in vivo* validation of the doxycycline chemogenetic switch **(a)** Representative images of U2OS cells at different time points following infection *at* MOI 0.01 with VV-TetR-iGFPLuc in the presence of 100 nM Dox. BFP signal, under continuous expression from vaccinia, indicates the presence of virus. **(b)** Representative brightfield and fluorescent images of different cell lines after infection at MOI 0.1 with VV-TetR-iGFPLuc in the absence or presence of 100 ng/ml Dox. **(c)** When comparing the expression pattern of GFPLuc directly transcribed by the wild-type vaccinia virus promoter with the doxycycline-controlled promoter, comparable levels of expression were observed between the two promoters in the presence of Dox. In some samples, Dox was added at the beginning of the experiment. **(d, e)** Multistep growth curves of VV-TetR-iGFPLuc compared to the control vaccinia virus at different time points from U2OS and A549 cells infected at MOI 0.01 in the presence or absence of 100 ng/ml Dox. **(f)** Quantitation of luciferase signal (RLU) from U2OS cells after 24 hours of infection with VV-TetR-iGFPLuc in the presence of different concentrations of Dox. **(g)** IVIS imaging 24 hours post viral infection and drug administration. HT-29 tumors were infected with VV-TetR-iGFPLuc at 1E7 PFU/tumor and were treated with 1 mg/kg of Dox. **(h)** Luciferase signal was quantified at various timepoints from HT-29 tumors infected with VV-TetR-iGFPLuc (1E7 PFU/tumor) following introduction of Dox into mice diets. Mice were given the Dox diet either 2 days post viral infection (Group 1, blue bars) or immediately following viral infection (Group 2, brown bars). For Group 2, Dox was removed from the diet after 2 days. Data shows the average total luciferase signal emitted from the tumor area. **(i)** IVIS imaging of HT-29 tumors following infection with VV-GFPLuc (1E7 PFU/tumor). Dox was introduced into the diet according to the schedule shown and groups assigned as described in panel G. **(j)** Quantitation of luciferase signal (measured as RLU) from HT-29 tumors following VV-GFPLuc infection and administration of Dox. Data shows the average total luciferase signal emitted from the tumor area. Scale bars = 40 µm in A & B. Data indicate means ± SD of three to five biological replicates. ns $P > 0.05$, *$P < 0.05$ **$P < 0.003841$, ***$P < 0.000125$, ****$P < 0.001$ in unpaired two-samples t-test.

Supplementary Figure 3

a

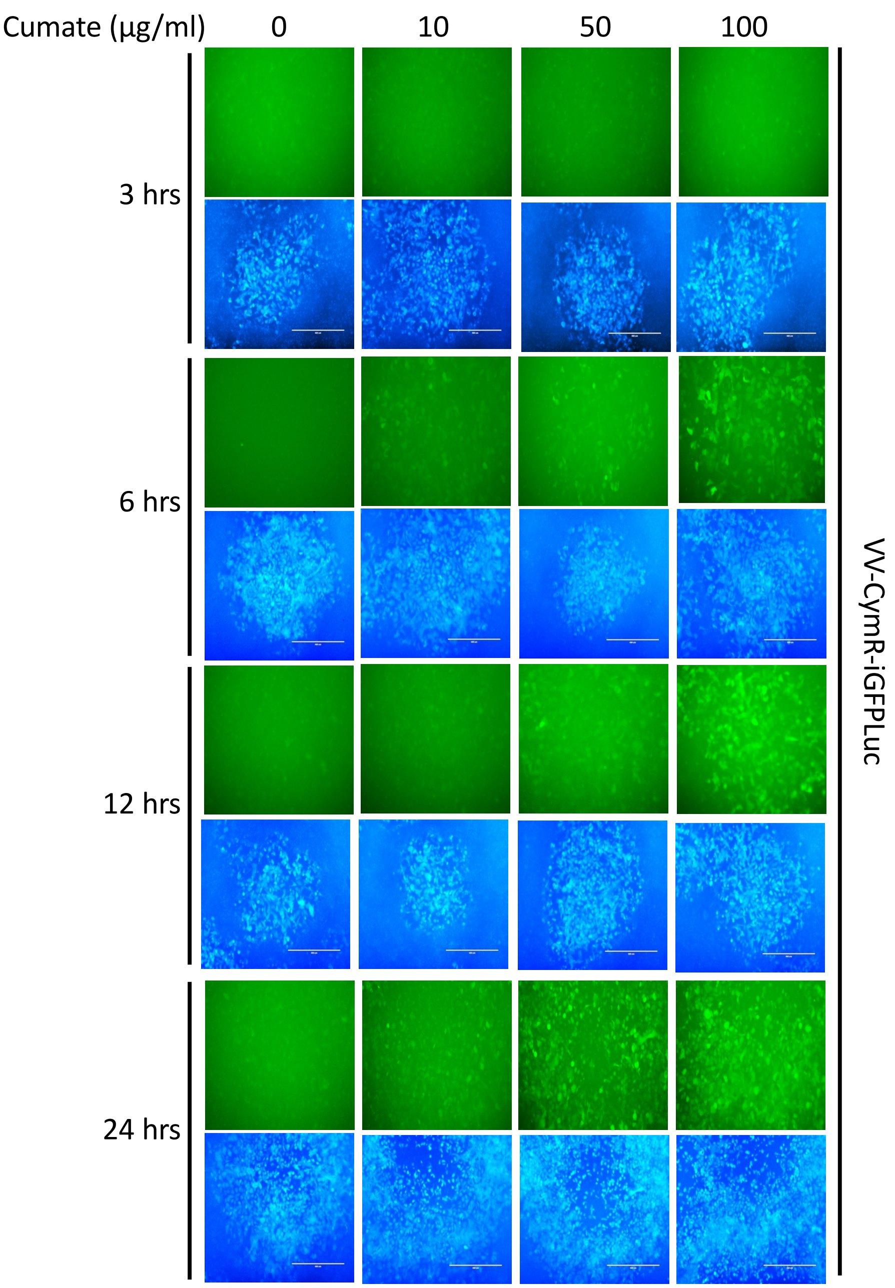

b

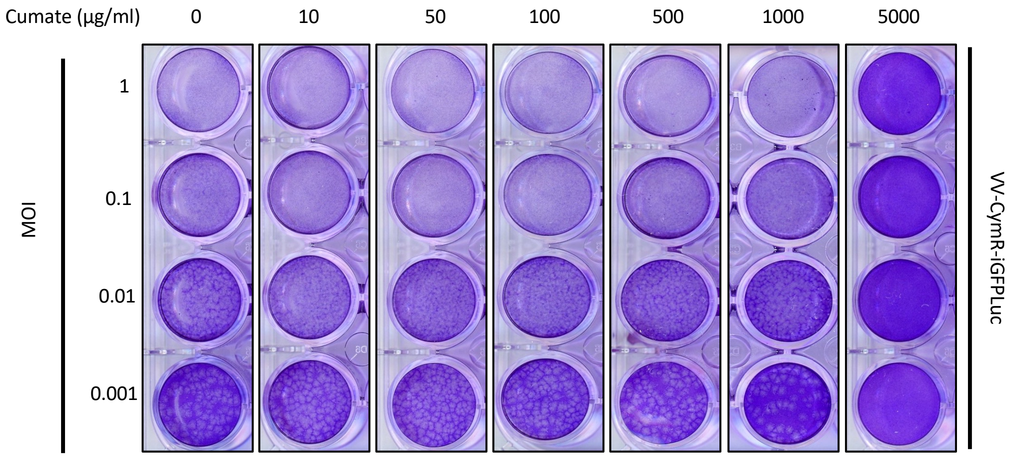

Continuation of Supplementary Figure 3

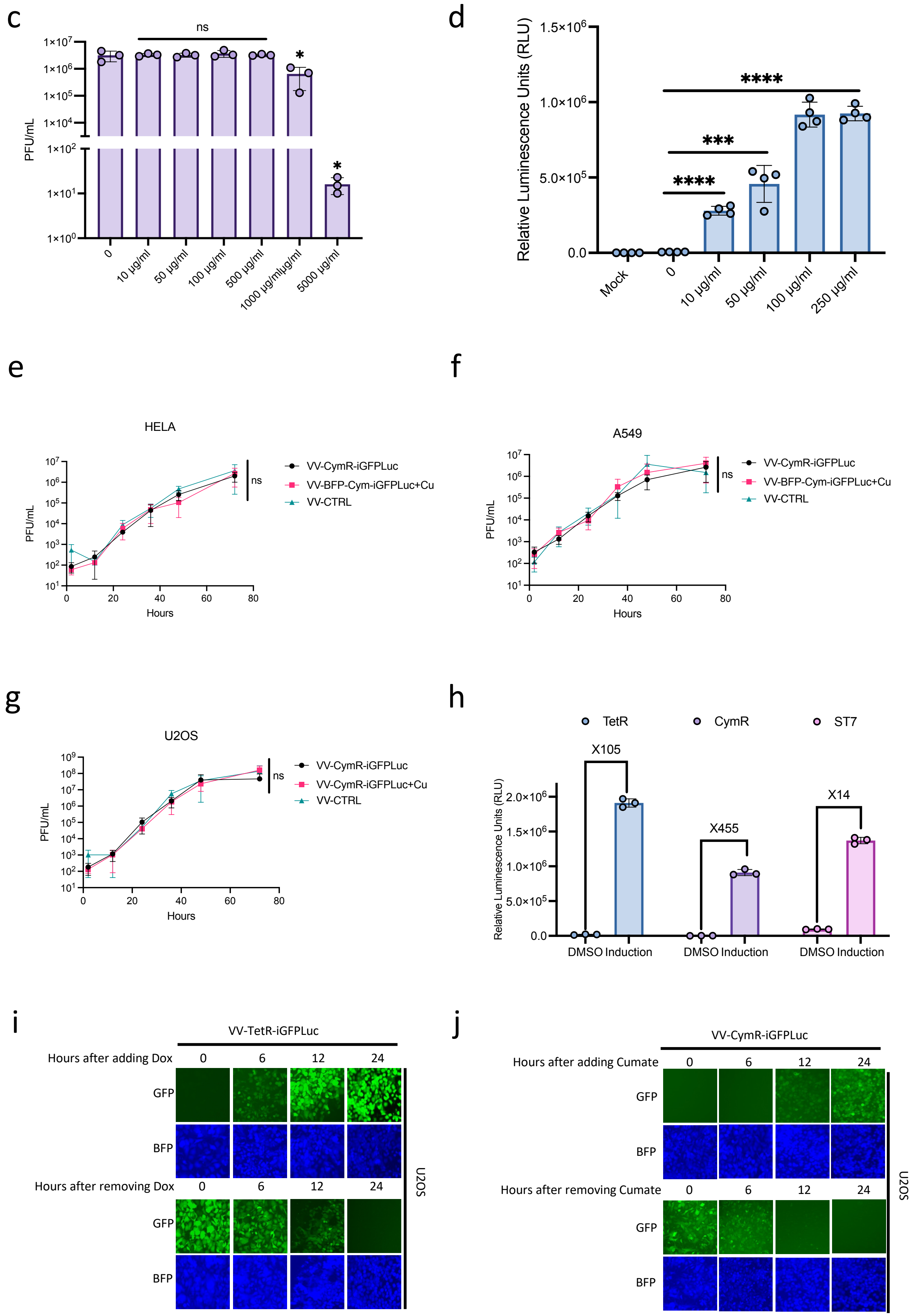

Continuation of Supplementary Figure 3

k

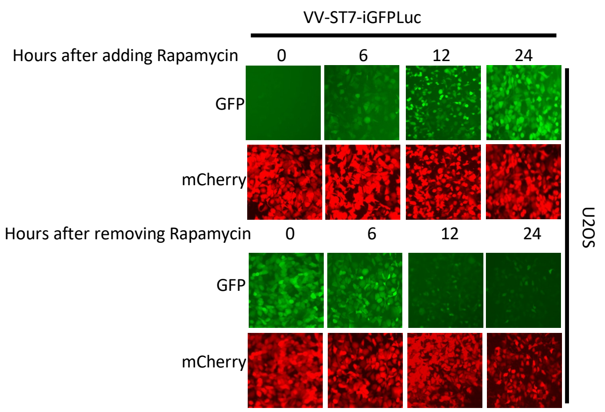

l

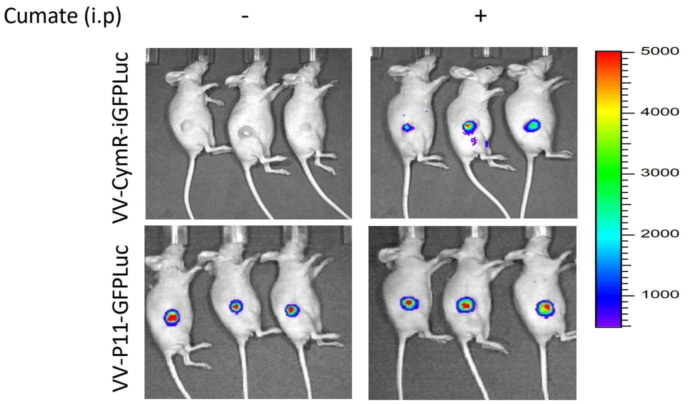

m

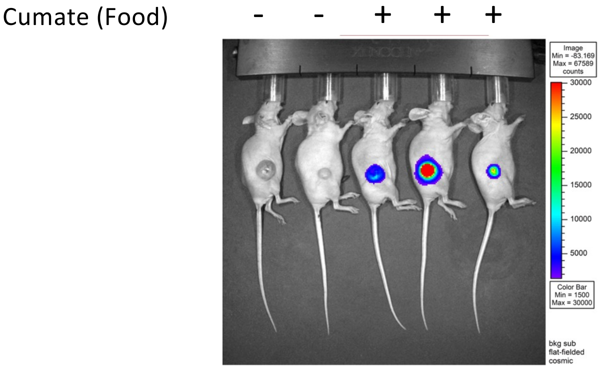

n

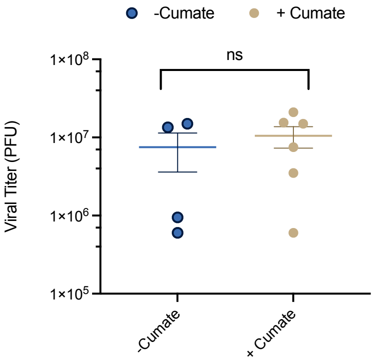

o

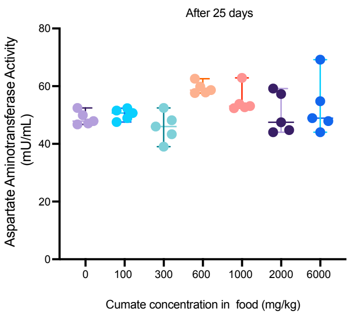

p

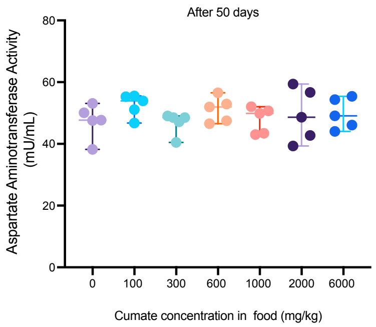

q

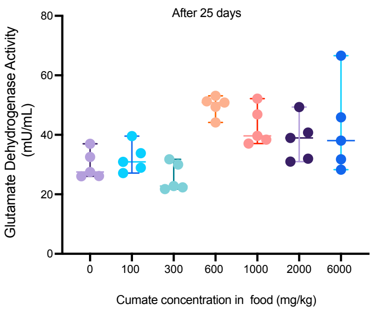

r

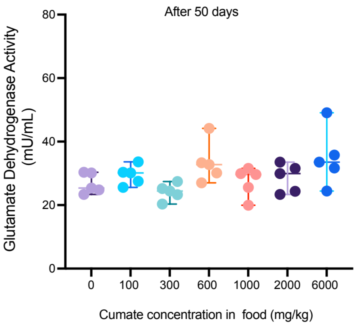

Supplementary Fig 3. Optimization of the CymR switch and comparison to ST7 and TetR systems. (a) Representative fluorescent images of U2OS cells at different time points after infection with VV-CymR-iGFPLuc (MOI 0.1) in the presence of different cumate concentration. BFP signal indicates viral infection. **(b, c)** U2OS cells were infected with VV-CymR-iGFPLuc at different MOIs in the presence of various concentrations of cumate for 24 hours. Cells were stained with crystal violet to detect viral plaques and viral load was determined for each group. **(d)** Luciferase activity was quantified in U2OS cells 24 hours following infection with VV-CymR-iGFPLuc in the presence of different concentrations of cumate. **(e-g)** Multistep growth curves of VV-CymR-iGFPLuc compared to the control vaccinia virus at different time points using Hela, A549, and U2OS cells infected at MOI 0.01 in the presence or absence of 100 ug/ml cumate. **(h)** Luciferase signal was quantified in U2OS cells 24 hours after infection with either VV-TetR-iGFPLuc, VV-CymR-iGFPLuc, or VV-ST7-iGFPLuc at an MOI 0.1 with or without induction. **(i-k)** Representative fluorescence images of U2OS cells infected with either VV-TetR-iGFPLuc, VV-CymR-iGFPLuc, or VV-ST7-iGFPLuc (MOI 0.1) and induced with Dox (100 ng/ml), cumate (100 ug/ml) or rapamycin (10 nM), respectively. Images were acquired at multiple time points and depict the presence of the inducible GFP fusion protein and viral infection (BFP or mCherry). **(l-n)** IVIS imaging and quantitation of viral load in HT-29 tumors injected with VV-GFPLuc or VV-CymR-iGFPLuc (1E7 PFU/tumor). When tumors reached ~150 mm^3 in size, cumate was injected intraperitoneally (1 mg/kg) or administered via diet (6000 mg/kg). **(o-r)** C57BL/6 mice were fed a cumate diet with varying amounts of cumate. Serum was collected after 25 days, and 55 days post treatment and quantitation of Aspartate Aminotransferase and Glutamate Dehydrogenase activity by ELISA was performed. Scale bars = 40 µm in panel B. Data indicate means ± SD of three to five biological replicates. ns P > 0.05, *P < 0.05 **P < 0.003841, ***P < 0.000125, ****P < 0.001 in unpaired two-samples t-test.

Supplementary Figure 4

a

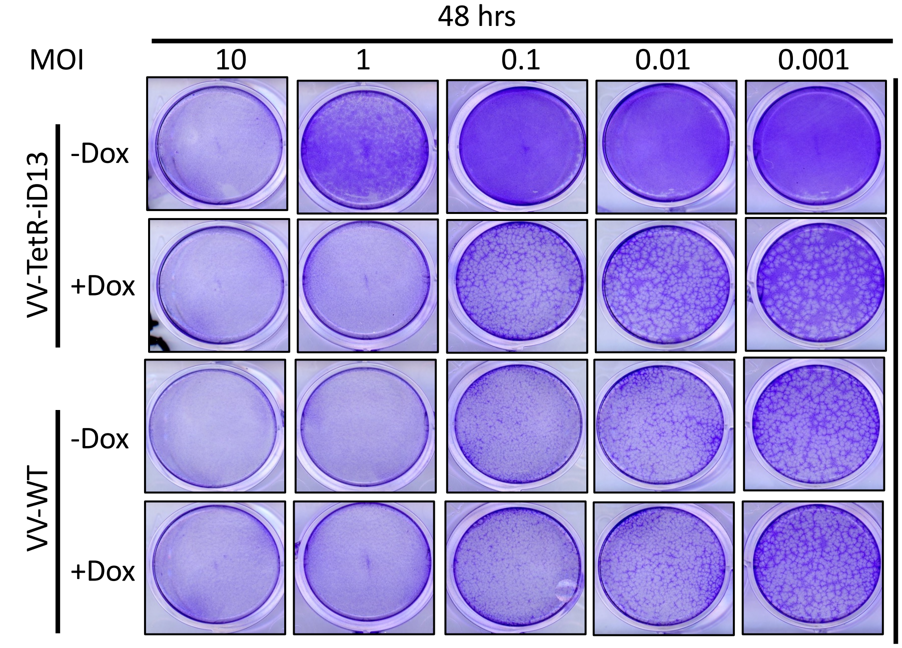

b

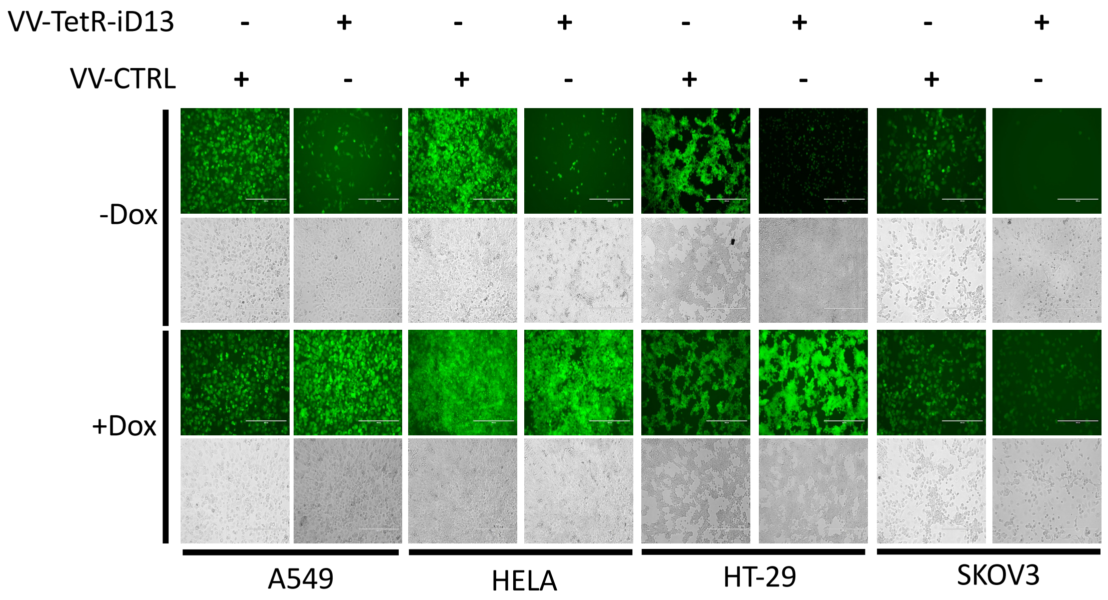

c

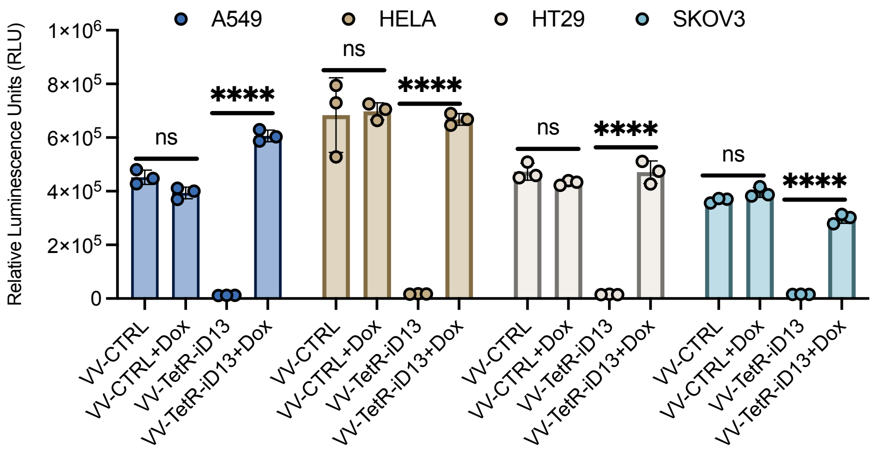

d

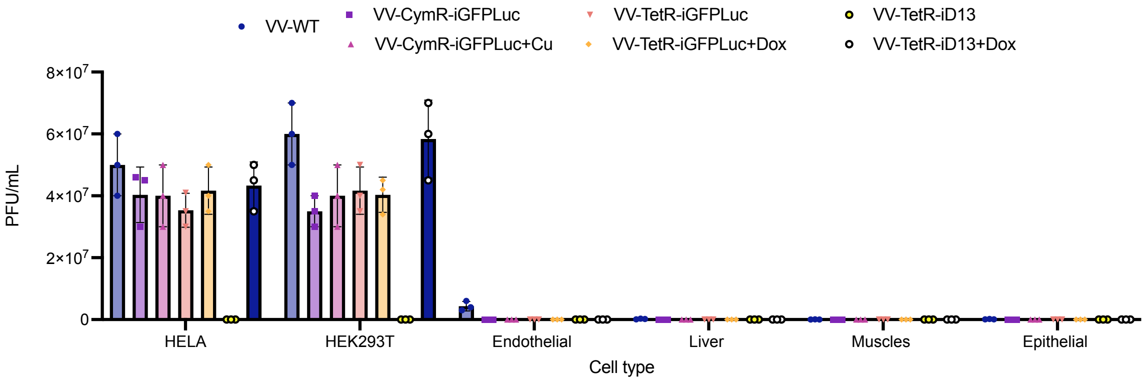

Supplementary Figure 4. *In vitro* validation of the conditional growth of the doxycycline inducible vaccinia virus (a) U2OS cells were infected at different MOIs with VV-TetR-iD13 or control vaccinia virus in the presence or absence of 100 ng/ml Dox. After 48 hours, plates were stained with crystal violet and representative pictures were taken. **(b, c)** A549, Hela, HT29, and SKOV3 cells were infected with control vaccinia virus or VV-TetR-iD13 at MOI 0.01 in the absence or presence of Dox at 100 ng/ml. After 48 hours, representative brightfield and fluorescent images were taken. Quantification of luciferase signal (RLU) is shown. **(d)** the viruses with chemogenic switch replicate 100 to 1000 times less efficiently in human primary cells when compared to cancer cells. Scale bars = 100 µm in panel B. Data indicate means ± SD of three to five biological replicates. ns $P > 0.05$, *$P < 0.05$ **$P < 0.003841$, ***$P < 0.000125$, ****$P < 0.001$ in unpaired two-samples t-test.

Supplementary Figure 5

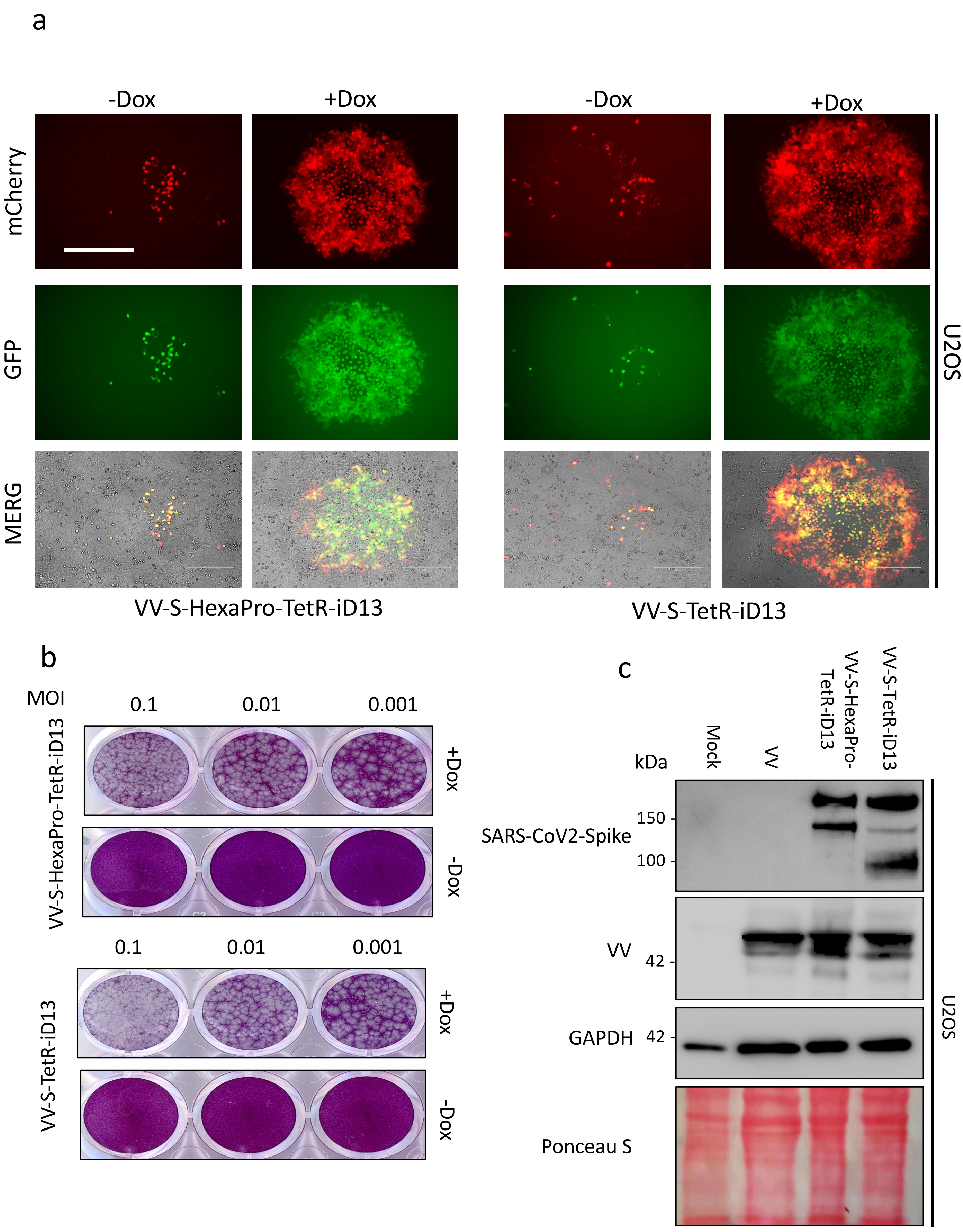

Supplementary Figure 5. ***In vitro*** **validation of the growth and antigen expression of the vaccine candidates. (a)** Images of U2OS cells infected with either VV-S-TetR-iD13 or VV-S-HexaPro-TetR-iD13 (MOI 0.01), in the presence or absence of 100 ng/ml Dox after 48 hours. The bottom row represents overlays of the red, green and brightfield channels. **(b)** U2OS cells were infected at different MOIs with either VV-S-TetR-iD13 or VV-S-HexaPro-TetR-iD13 in the presence or absence of 100 ng/ml Dox. After 48 hours, plates were stained with crystal violet and representative pictures were taken. **(c)** U2OS cells were infected with control or vaccine viruses at MOI 1 and samples were lysed and collected after 24 hours. Western blotting was performed to detect SARS-CoV2 Spike and vaccinia virus. Scale bars = 200 µm in panel A. Data indicate means ± SD of three to five biological replicates. ns $P > 0.05$, *$P < 0.05$ **$P < 0.003841$, ***$P < 0.000125$, ****$P < 0.001$ in unpaired two-samples t-test.

Supplementary Figure 6

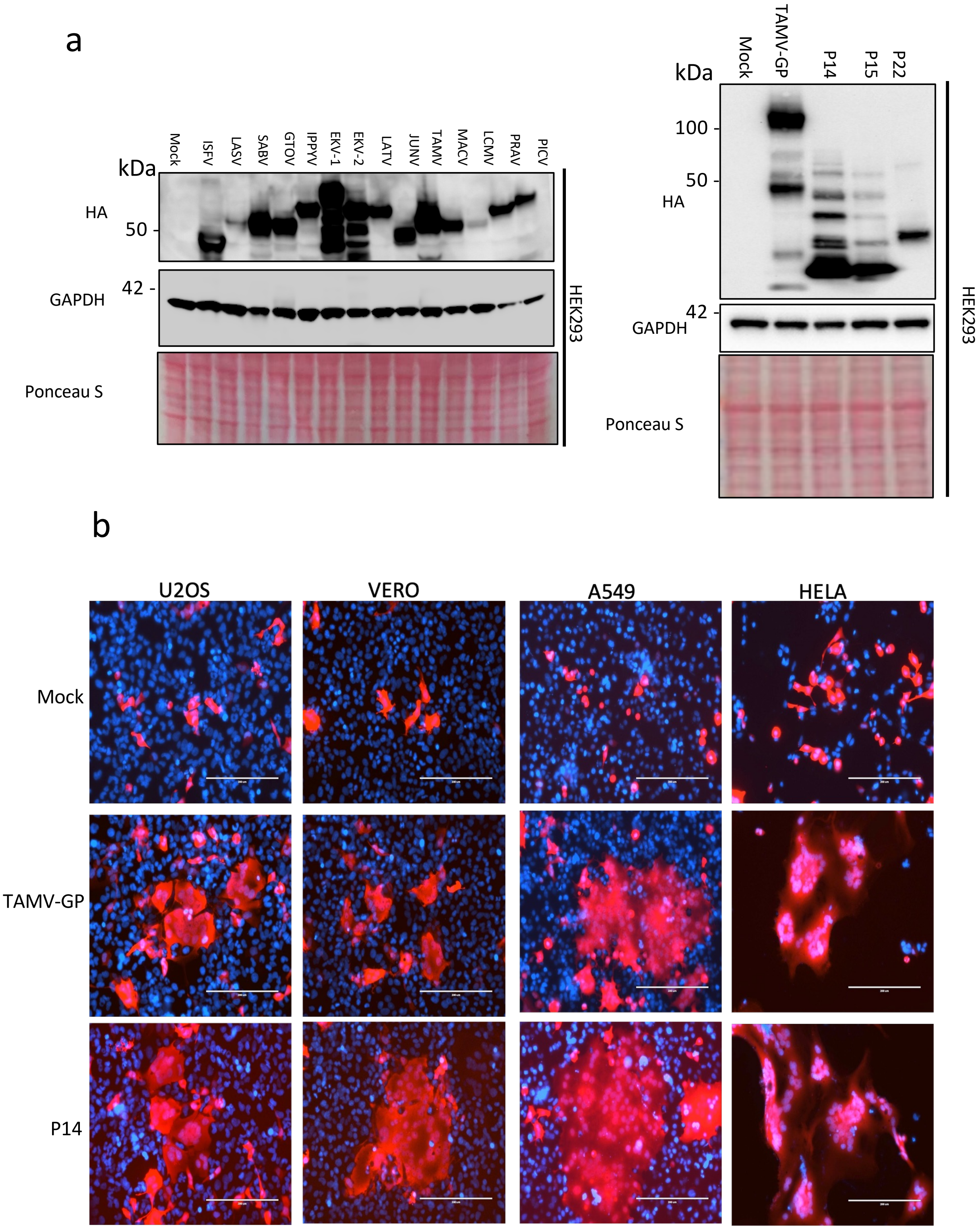

Continuation of Supplementary Figure 6

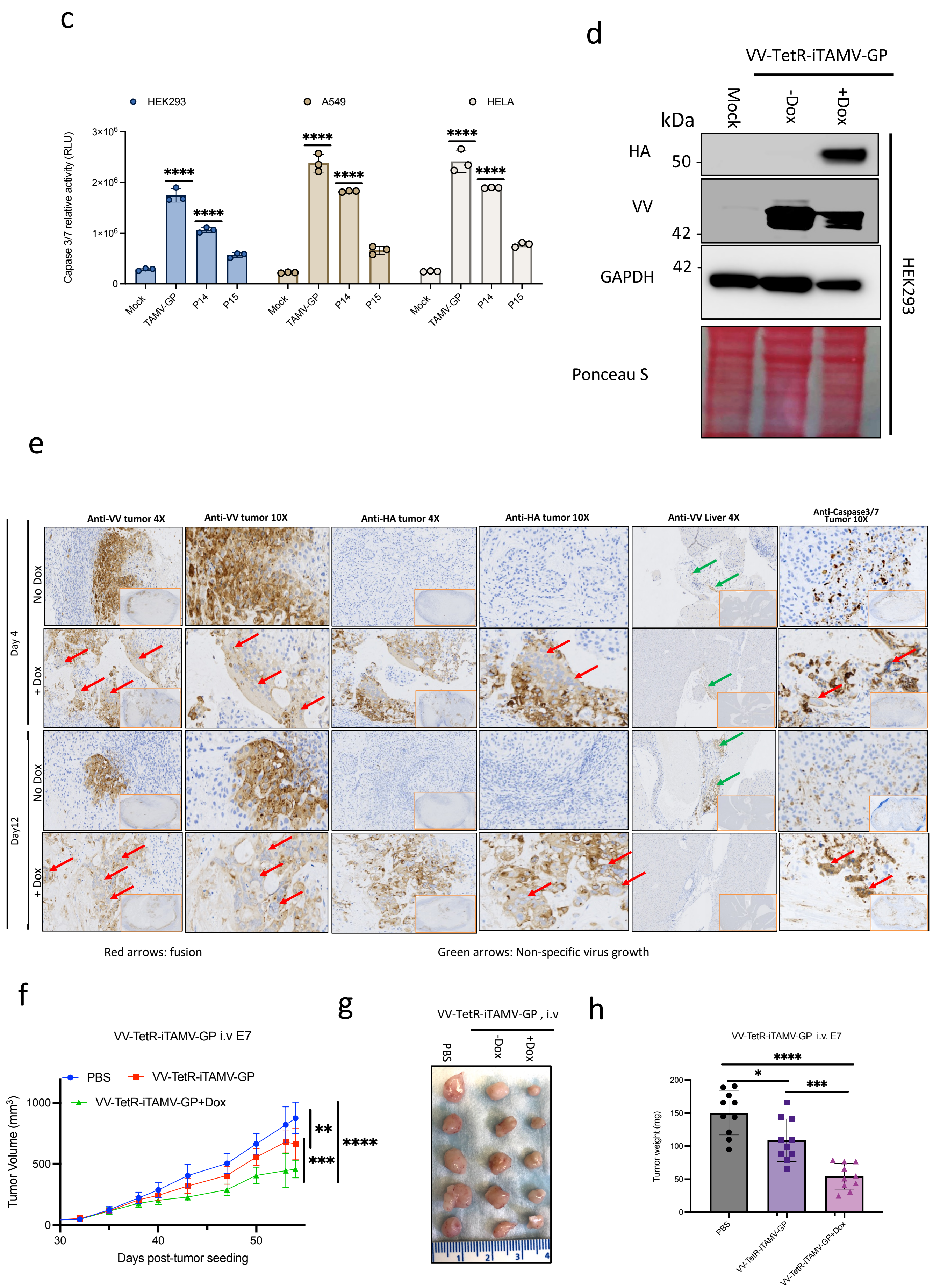

Supplementary Figure 6. ***In vitro*** **validation and** ***in vivo*** **application of conditionally expressed TAMV-GP (a)** Western blotting analysis of HEK293 cells transfected with different arenavirus and reovirus glycoproteins. All glycoproteins expressed a HA tag for detection. **(b)** Representative fluorescent images of U2OS, Vero, A549, and Hela cells 24 hours following infection with TAMV-GP and P14 to show syncytia. **(c)** Quantitation of caspase 3/7 activity in HEK293, A549, and Hela cells 24 hours following transfection with TAMV-GP, P14, and P15. **(d)** Western blotting analysis of HEK293 cells infected with VV-TetR-iTAMV-GP (MOI 0.1) tagged with HA in the presence or absence of Dox at 100 ng/ml. **(e)** Full immunohistochemistry staining shown in Figure 7I. **(f-h)** Mice harboring A549 tumors were intravenously injected with VV-TetR-iTAMV-GP (1E7 PFU/mice) when tumors reached ~150 mm^3 in size and depending on the treatment group received 625 mg/kg of Dox in their diet two days after virus injection. Tumor volume was measured at regular intervals **(f)**. **(g-h)** Tumors were harvested after 60 days and weighed. Scale bars = 200 µm in panel B. Data indicate means ± SD of three to five biological replicates. ns $P > 0.05$, $*P < 0.05$ $**P < 0.003841$, $***P < 0.000125$, $****P < 0.001$ in unpaired two-samples t-test.

Supplementary Figure 7

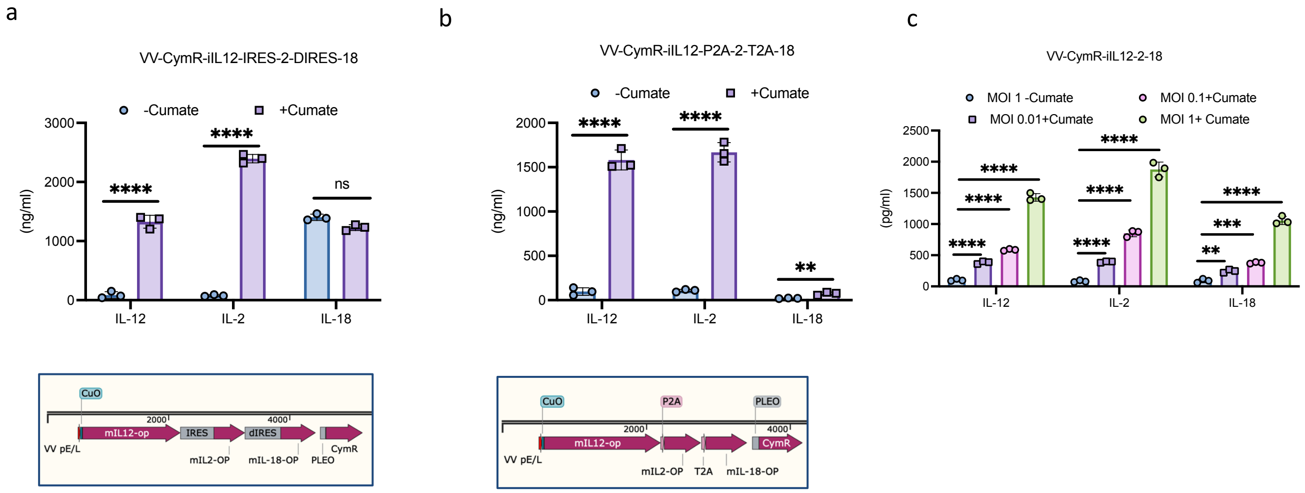

Supplementary Figure 7. Evaluating the optimal design for tandem expression of three interleukins controlled by a cumate switch. (a-b) Schematic representation of the constructs and ELISA analysis of IL-2, IL-12, IL-18 concentrations from supernatants of U2OS cells 6 hours after infection with VV-CymR-iIL12-IRES-2-IRES-18 **(a)** or VV-CymR-iIL12-2-18 **(b)**. Cells were infected at an MOI of 1 and treated with 100 ug/ml cumate or PBS. **(**C) ELISA analysis of IL-2, IL-12, IL-18 from supernatants of U2OS cells 6 hours after infection with VV-CymR-iIL12-2-18 at different MOIs and treatment with 100 ug/ml cumate or PBS. Scale bars = 40 µm in panel B. Data indicate means ± SD of three to five biological replicates. ns P > 0.05, *P < 0.05 **P < 0.003841, ***P < 0.000125, ****P < 0.001 in unpaired two-samples t-test.

Supplementary Figure 8

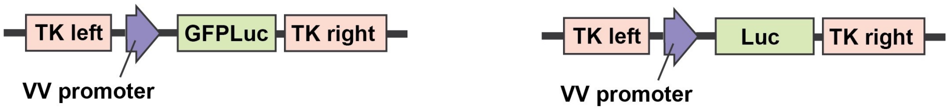

Supplementary Figure 8. Schematic illustration of VV-CTRL (left), VV-GFPLuc (right).

Supplementary Figure 9- Uncropped western blots are shown thereafter.

Supplementary Figure 5C

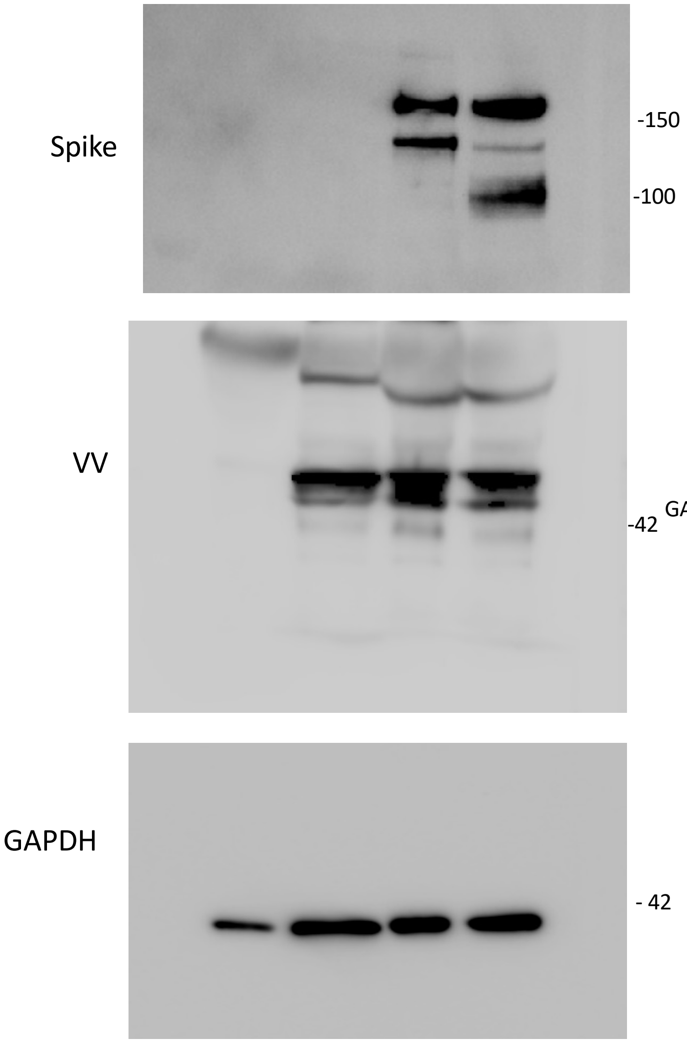

Supplementary Figure 6A

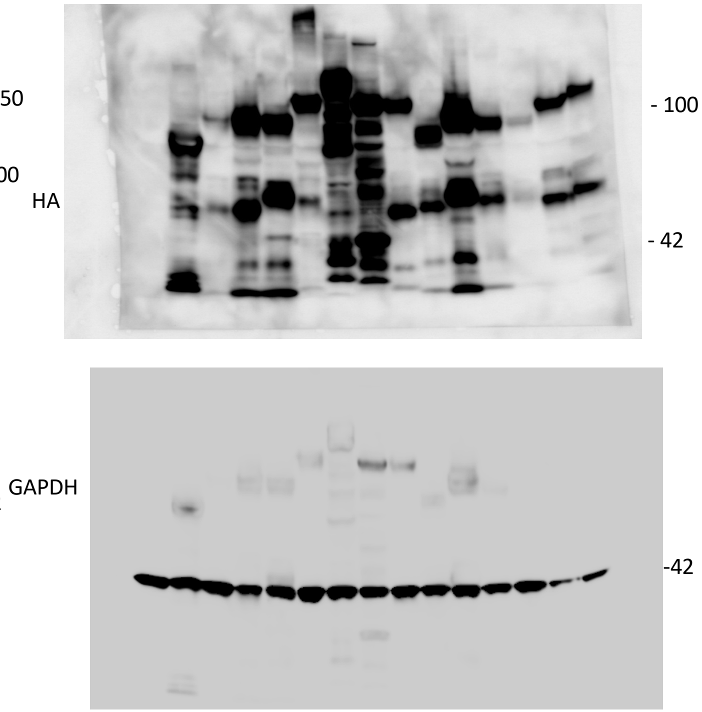

Supplementary Figure 6D

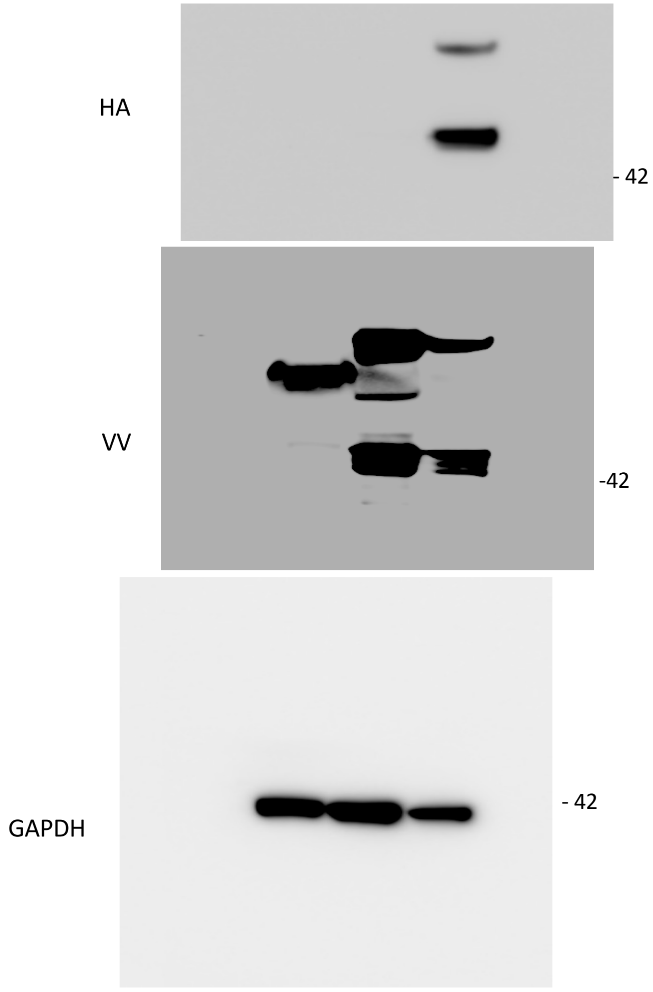

Supplementary Figure 6A

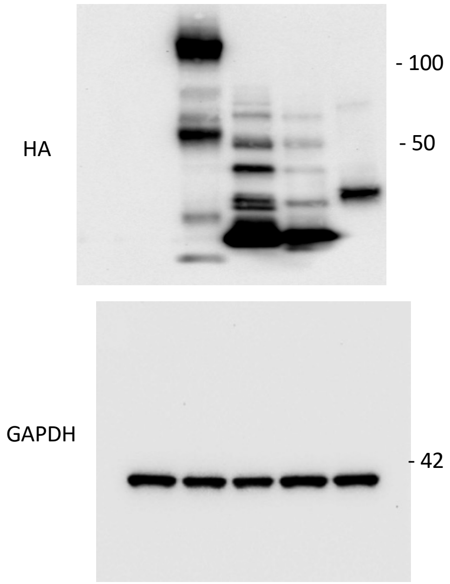

Supplement: Supplementary file 1 — Supplementary Information [file 41467_2023_38651_MOESM1_ESM.pdf]
