# Supplementary material for: Synthetic virology approaches to improve the safety and efficacy of oncolytic virus therapies
Source: Nat Commun. 2023 May 26;14:3035. doi: 10.1038/s41467-023-38651-x (PMC10213590; doi:10.1038/s41467-023-38651-x)
Supplement: Supplementary file 2 — Reporting Summary [file 41467_2023_38651_MOESM2_ESM.pdf]

nature portfolio

Corresponding author(s): John C. Bell

Last updated by author(s): May 2, 2023

Reporting Summary

Nature Portfolio wishes to improve the reproducibility of the work that we publish. This form provides structure for consistency and transparency in reporting. For further information on Nature Portfolio policies, see our Editorial Policies and the Editorial Policy Checklist.

Statistics

For all statistical analyses, confirm that the following items are present in the figure legend, table legend, main text, or Methods section.

n/a	Confirmed	
[ ]	[x]	The exact sample size (*n*) for each experimental group/condition, given as a discrete number and unit of measurement
[ ]	[x]	A statement on whether measurements were taken from distinct samples or whether the same sample was measured repeatedly
[ ]	[x]	The statistical test(s) used AND whether they are one- or two-sided<br>*Only common tests should be described solely by name; describe more complex techniques in the Methods section.*
[x]	[ ]	A description of all covariates tested
[ ]	[x]	A description of any assumptions or corrections, such as tests of normality and adjustment for multiple comparisons
[ ]	[x]	A full description of the statistical parameters including central tendency (e.g. means) or other basic estimates (e.g. regression coefficient) AND variation (e.g. standard deviation) or associated estimates of uncertainty (e.g. confidence intervals)
[ ]	[x]	For null hypothesis testing, the test statistic (e.g. *F*, *t*, *r*) with confidence intervals, effect sizes, degrees of freedom and *P* value noted<br>*Give P values as exact values whenever suitable.*
[x]	[ ]	For Bayesian analysis, information on the choice of priors and Markov chain Monte Carlo settings
[x]	[ ]	For hierarchical and complex designs, identification of the appropriate level for tests and full reporting of outcomes
[x]	[ ]	Estimates of effect sizes (e.g. Cohen's *d*, Pearson's *r*), indicating how they were calculated

Our web collection on statistics for biologists contains articles on many of the points above.

Software and code

Policy information about availability of computer code

Data collection	Data collection, such as images ,etc., were done with specific softwares installed on instruments and as detailed in the Methods section. qPCR data was collected using the Rotor-Gene RG-3000A (Corbett Research) and the Rotor-Gene 6.1.81 Software or the 7500 Fast Real-Time PCR System (Applied Biosystems) and the 7500 Software v2.3. DNA and RNA gels were imaged on the Epi Chemi II Darkroom Imager (UVP) using the LabWorks software version 4.6. Immunohistochemistry pictures were acquired using the Scanscope (Aperio) and ImageScope version 11.1.2.760 software (Aperio). The alamarBlue cell viability assay samples were read on the Fluoroskan Ascent FL plate reader (Thermo Fischer Scientific) using the Ascent Software Version 2.6. The crystal violet lift assay samples were read on the Multiskan Ascent plate reader (Thermo Fischer Scientific) using the Ascent Software Version 2.6. Statistical data analysis was performed using GraphPad Prism (version 9.3.1).
Data analysis	For the RNA sequencing analysis, adapters were trimmed using Trimmomatic (version 0.22, Bolger et al., 2014). Transcript abundance and differential expression analysis were performed using cufflinks and cuffdiff (version 2.2.1, Trapnell et al., 2010) as part of a previously described pipeline (Trapnell et al., 2012). Genes with an FDR value smaller than 0.05 and with a fold change greater or smaller than 4 were considered for pathway analysis using gProfiler (version 0.2.3, Reimand et al., 2007). Heat maps from RNA sequencing data were generated using Package 'pheatmap'- R Project version 1.0.8.

For manuscripts utilizing custom algorithms or software that are central to the research but not yet described in published literature, software must be made available to editors and reviewers. We strongly encourage code deposition in a community repository (e.g. GitHub). See the Nature Portfolio guidelines for submitting code & software for further information.

Data

Policy information about availability of data

All manuscripts must include a data availability statement. This statement should provide the following information, where applicable:

- Accession codes, unique identifiers, or web links for publicly available datasets
- A description of any restrictions on data availability
- For clinical datasets or third party data, please ensure that the statement adheres to our policy

Uncropped western blots shown in supplementary figures are displayed in Supplementary Figure 9.

Human research participants

Policy information about studies involving human research participants and Sex and Gender in Research.

Reporting on sex and gender	N/A
Population characteristics	N/A
Recruitment	N/A
Ethics oversight	N/A

Note that full information on the approval of the study protocol must also be provided in the manuscript.

Field-specific reporting

Please select the one below that is the best fit for your research. If you are not sure, read the appropriate sections before making your selection.

☒ Life sciences ☐ Behavioural & social sciences ☐ Ecological, evolutionary & environmental sciences

For a reference copy of the document with all sections, see nature.com/documents/nr-reporting-summary-flat.pdf

Life sciences study design

All studies must disclose on these points even when the disclosure is negative.

Sample size	No particular statistical method was used to predetermine sample size. Sample size was determined based on previous experiments performed in our hands that gave statistically significant data (Breitbach, et al., Molecular Therapy [2011]; Ilkow et al., Nature Medicine [2015]; Bourgeois-Daigneault et al., Science Translational Medicine [2018]) and standards in the field of research. Typically, cell culture experiments were performed using three or more independent samples while animal experiments were performed with 5-10 mice/group. The low variability within the same experimental condition/s group, indicated by SEM or SD, confirms that the selected sample size is appropriate to detect statistical differences (if present) among treatment conditions. The nature of the n is indicated in details in the methods section and in each Figure and Supplementary Figure legend.
Data exclusions	In certain rare cases, outliers were excluded of sample analysis if they were clearly abnormal or due to technical experimental malfunctions during sample preparation. Exclusion criteria was pre-established. Animals that did not develop tumours by the time of treatment, where excluded from the study.
Replication	All in vitro experiments were successfully repeated at least twice unless otherwise stated in the manuscript. Unless otherwise stated in the figure legend, mouse studies were performed once to minimize the use of animals. RNA seq experiments were only perform using two technical replicates due to high experimental cost. However, relevant hits were further validated using different techniques.
Randomization	In all cell culture experiments, wells or plates were randomly assigned to the different treatment/condition group. For in-vivo studies, Animal cohorts were randomized following tumour implantation before initiation of treatment plan. Mean tumor size per group was also verified for experiments with subcutaneous tumours to ensure comparable tumour size between groups at treatment initiation.
Blinding	For in-vitro studies data acquisition and/or analyses was conducted blinded. Animals experiments were not performed blinded since cages with animals treated with oncolytic viruses needed to be identified at the animal facility. However, the operator that performed the treatments and tumour measurements was blinded to the allocation group. The person performing the treatments, measuring the tumours, etc., and analyzing the data was unaware or the treatment group.

Reporting for specific materials, systems and methods

We require information from authors about some types of materials, experimental systems and methods used in many studies. Here, indicate whether each material, system or method listed is relevant to your study. If you are not sure if a list item applies to your research, read the appropriate section before selecting a response.

Materials & experimental systems

n/a	Involved in the study
☐	☒ Antibodies
☐	☒ Eukaryotic cell lines
☒	☐ Palaeontology and archaeology
☐	☒ Animals and other organisms
☒	☐ Clinical data
☒	☐ Dual use research of concern

Methods

n/a	Involved in the study
☒	☐ ChIP-seq
☒	☐ Flow cytometry
☒	☐ MRI-based neuroimaging

Antibodies

Antibodies used

The following antibodies SARS-CoV-2 Spike (GeneTex, 1A9, Cat. No. GTX632604), HA (Thermo Fisher Scientific, 26183), VV (Abcam, ab117453), GADPH (Cell Signaling, 2118L), monoclonal RBD (Cat. No. MBS434247) antibodies, which were used at a dilution of 1:1000. Cell Signaling supplied HRP conjugated antimouse (7076S) and anti-rabbit (7074S) IgG secondary antibodies, which were used at a dilution of 1:2000.

Validation

All primary antibodies used in this project were obtained from commercial suppliers, which have validated the specificity of the antibodies by protein knockout/knockdown approaches, using protein overexpression or by induction of protein expression. All antibodies have been extensively used and cited by other research groups. Detailed validation information as obtained from the supplier's data sheet is summarized below:
SARS-CoV-2 Spike (GeneTex, 1A9, Cat. No. GTX632604):
This antibody has been extensively cited and has been valided for WB, ELISA, and IF by the manufacturer.
HA (Thermo Fisher Scientific, 26183):
This antibody has been extensively cited and has been validated by Thermo Fisher Scientific, the details of which can be found on their website.
VV (Abcam, ab117453) used for Immunohistochemistry:
This antibody has been tested by immunohistochemical analysis of paraffin-embedded mouse tumor tissue and mouse liver tissue.
GADPH (Cell Signaling, 2118L):
This antibody has been extensively cited and has been valided for WB by the manufacturer.
monoclonal RBD (Cat. No. MBS434247):
This antibody has been extensively cited and has been valided for ELISA by the manufacturer and the details of its titration can be found on their website.

Eukaryotic cell lines

Policy information about cell lines and Sex and Gender in Research

Cell line source(s)

All cell lines were purchased from the American Type Culture Collections (Manassas, VA). The cells were grown in Dulbecco's Modified Eagle Medium/DMEM (GE Healthcare Life Sciences; ON, CAN) or Roswell Park Memorial Institute/RPMI 1640 Medium (Gibco; MA, USA), supplemented with 10% fetal bovine serum/FBS (Gibco). Cells were kept at 37°C in a humidified environment with 5% CO2. Cells co-cultured with effector cells were cultivated in RPMI medium supplemented with 10% FBS and 1% penicillin/streptomycin (volume/volume) (Gibco). Co-cultures were kept at 37°C in a humidified environment with 5% CO2. Cells were regularly checked for mycoplasma contamination using PCR (e-Myo VALiD Detection Kit, 25239, LiliF Diagnostics; South Korea) and were shown to be mycoplasma-free.

Authentication

All cell lines were recently purchased from renowned biorepositories. Although cell lines obtained from biorepositories have not been authenticated upon receiving, they appeared to be authentic based on their in vivo and in vitro growth. Primary cell lines were not authenticated.

Mycoplasma contamination

All cell lines were subjected to routine mycoplasma contamination testing using Hoechst stain (Invitrogen) and the e-Myco VALiD Myco PCR detection kit (FroggaBio). All cells lines have tested negative for mycoplasma contamination.

Commonly misidentified lines
(See ICLAC register)

No commonly misidentified cell lines were used in this study.

Animals and other research organisms

Policy information about studies involving animals; ARRIVE guidelines recommended for reporting animal research, and Sex and Gender in Research

Laboratory animals	All mice used in this study were 6-8 week old female CD1 nude and C57BL/6 mice purchased from Charles River or Jackson Laboratories, as described in the Methods section. All mice were housed in temperatures 20-25 °C, humidity 20-70% and a 12 h light/ 12 h dark cycle.
Wild animals	No wild animals were used in this study.
Reporting on sex	This study applies to both sexes.
Field-collected samples	This study did not include samples collected from the field.
Ethics oversight	The study discussed in this document adheres to all pertinent ethical guidelines at OHRI and the University of Ottawa (certificate for biohazardous material utilization GC317-125-12). All samples from patients were acquired via the Global Tissue Consenting committee at OHRI. The University of Ottawa's institutional animal care committee approved all animal experiments (Protocol ID: OHRI2870 and MEe-2258) which were conducted following the National Institutes of Health and the Canadian Council on Animal Care standards.

Note that full information on the approval of the study protocol must also be provided in the manuscript.
